# Caatinga diaspores: Descriptive overview of dispersal units of seasonally dry tropical forests and woodlands

**DOI:** 10.1002/ecy.70024

**Published:** 2025-02-25

**Authors:** Fabricio Francisco Santos da Silva, Edjane Silva Damasceno, Ramon Athayde de Souza Cavalcante, Francinete Alves do Nascimento, Mateus Brandão Prates, Luís Francisco Mello Coelho, Daniel Salgado Pifano, Renato Garcia Rodrigues

**Affiliations:** ^1^ Centre for Ecology and Environmental Monitoring (NEMA) University of Vale do São Francisco (UNIVASF) Petrolina Pernambuco Brazil; ^2^ Center for Research in Plant Biology (CEBIVE) University of Vale do São Francisco (UNIVASF) Petrolina Pernambuco Brazil

**Keywords:** endemism, *ex situ*, SDTFW, seed collection, seed dormancy, seed longevity, seed mass, seed shape, seed storage, seed traits

## Abstract

Dispersal unit characteristics provide crucial insights into species ecology and are essential for the conservation and restoration of ecosystems. The Caatinga, the largest ecosystem of seasonally dry tropical forests and woodlands in South America, remains underrepresented in terms of dispersal unit data, which are often scattered across the scientific literature or remain unpublished. To address this gap, we compiled a dataset of morphophysiological data for 100 native taxa, including key information such as germination, seed water content, 1000 seed mass, fruit shape, and the geographic coordinates of 1981 seed lots. Over nine years, more than 60 t of dispersal units were harvested, representing 91% of the most dominant woody species in this ecosystem. These records stem from environmental licensing actions associated with the São Francisco River Integration Project (PISF), the Re‐Habitar Ararinha Azul Project, and verified literature. This dataset, the first of its kind for the Caatinga, offers valuable potential for research on forest dynamics, dispersal, germination, conservation, and ecological restoration in the Brazilian semiarid region. We hope this data paper provides reliable information on local flora distribution, dispersal syndromes, and morphological descriptions, while also suggesting methods for overcoming seed dormancy in the Caatinga. No copyright restrictions apply to this dataset, but please cite this data paper in publications. We also encourage researchers and educators to inform us of how they are using the data.

## Resumo

As características das unidades de dispersão podem fornecer insights valiosos sobre a ecologia das espécies e suas implicações para a conservação e restauração de ecossistemas. A Caatinga é o maior ecossistema de Florestas e Bosques Tropicais Sazonalmente Secos da América do Sul. No entanto, as informações sobre as unidades de dispersão da região semiárida brasileira ainda são insuficientes e dispersas na literatura científica ou em dados não publicados. Para preencher essas lacunas e fornecer informações detalhadas sobre as unidades de dispersão da Caatinga, compilamos um conjunto de dados morfofisiológicos de 100 táxons nativos, incluindo informações como germinação, teor de água das sementes, peso de 1000 sementes, forma do fruto e coordenadas geográficas de 1981 lotes de sementes. No total, mais de 60 toneladas de unidades de dispersão foram coletadas, cobrindo 91% das espécies lenhosas mais dominantes neste ecossistema. Esses registros provêm de resultados parciais de ações de licenciamento ambiental do Projeto de Integração do Rio São Francisco com Bacias Hidrográficas do Nordeste Setentrional (PISF), do projeto RE‐Habitar Ararinha Azul e de literatura confiável. Nosso conjunto de dados é o primeiro do tipo para a Caatinga e tem potencial para uso em estudos sobre dinâmica florestal, dispersão, germinação, conservação e restauração ecológica da região semiárida brasileira. Portanto, esperamos que este artigo de dados possa oferecer informações valiosas e confiáveis sobre a distribuição da flora local, síndromes de dispersão, descrições morfológicas e sugerir diferentes métodos para superação de dormência de sementes na Caatinga. Não há restrições de direitos autorais associadas ao uso deste conjunto de dados. Pedimos, por favor, que citem este artigo de dados ao utilizar as informações em publicações. Também solicitamos que pesquisadores e educadores nos informem sobre como estão utilizando os dados.

## CONFLICT OF INTEREST STATEMENT

The authors declare no conflicts of interest.

## Supporting information


Appendix S1.

## Data Availability

The complete dataset is available as Data S1. Data are also available in Mendeley Data at https://doi.org/10.17632/8t6yh9rm26.2.

