# Supplementary material for: Caatinga diaspores: Descriptive overview of dispersal units of seasonally dry tropical forests and woodlands
Source: Ecology. 2025 Feb 25;106(2):e70024. doi: 10.1002/ecy.70024 (PMC11859873; doi:10.1002/ecy.70024)
Supplement: Supplementary file 1 — Appendix S1. [file ECY-106-e70024-s001.zip › MetadataS1.pdf]

Data Paper published in *Ecology*, an Ecological Society of America journal

Metadata S1

Caatinga diaspores: A descriptive overview of dispersal units of seasonally dry tropical forests and woodlands

Fabricio Francisco Santos da Silva; Edjane Silva Damasceno; Ramon Athayde de Souza Cavalcante; Francinete Alves do Nascimento; Mateus Brandão Prates; Luís Francisco Mello Coelho; Daniel Salgado Pifano; Renato Garcia Rodrigues

Introduction

Mapping the ecological attributes of dispersal units is a critical step in ecological conservation and restoration. These attributes provide essential characteristics for decision-making, including the careful selection of species for incorporation into ecological restoration projects (Shackelford *et al.* 2021; Carvalho *et al.* 2022; Laumann *et al.* 2023). The Caatinga represents the largest and most continuous area of seasonally dry tropical forest and woodland (SDTFW) in the Neotropics, harbouring a high diversity of species adapted to extreme dry conditions (DRYFLOR 2016; Queiroz *et al.* 2017; Fernandes and Queiroz 2018; Silva and Souza 2018), including many endemic species (Fernandes *et al.* 2022). However, climate change and human activities have threatened the survival of species crucial to the functioning of SDTFW (Dantas *et al.* 2020; Manhães *et al.* 2022). Given this, analysing a detailed data paper on Caatinga diaspores can offer valuable insights into the ecology of these species and their implications for the conservation and restoration of these ecosystems. This is particularly significant, as half of the original Caatinga coverage has already been lost, and the remaining areas continue to be impacted by chronic anthropogenic disturbances, with few areas protected by conservation units (Fonseca *et al.* 2017; Antongiovanni *et al.* 2018, 2020).

The Caatinga supports diverse ecological interactions driven by adaptive processes between plants and animals. These interactions, which are crucial for ecosystem maintenance, involve complex relationships with pollinators and dispersers. Plants exhibit specific morphological and phenological adaptations to these agents. Plants exhibit specific morphological and phenological adaptations to these agents. Seed dispersal by birds, mammals, and even the wind plays a vital role in distributing dispersal units, ensuring ecosystem regeneration and biodiversity (Meiado *et al.* 2012; Queiroz *et al.* 2017; Domingos-Melo *et al.* 2023). Of the 1,462 species recorded (Fernandes *et al.* 2020), 5.6% are woody species. These findings align with studies suggesting that a small percentage of species may dominate tropical forests (Cooper *et al.* 2024). Consistent with the results showing the 48 most common species in the Caatinga (Oliveira *et al.* 2020, 2022), our dataset stands out as one of the most comprehensive collections of morphophysiological data on dispersal units from the Caatinga, covering 91% of the most dominant woody species. To our knowledge, no other dataset of this scale and specificity exists for SDTFW in the Neotropics, making it a crucial resource for ecological research, restoration efforts, and conservation strategies.

Infrastructure and ecosystem restoration projects can provide valuable opportunities for biodiversity exploration and conservation. The Projeto de Integração do Rio São Francisco (PISF, São Francisco River Integration Project) and the Projeto Re-Habitar Ararinha Azul (Re-Habitar Ararinha Azul Project) exemplify this, offering a unique setting for conducting the most comprehensive seed sampling ever carried out in the Caatinga. The PISF is one of the largest water infrastructure projects globally, located in the Brazilian semi-arid region (MIDR 2023). However, the project has also created a significant environmental liability, necessitating the restoration of approximately 2,000 hectares of degraded land (NEMA 2023). Similarly, the Re-Habitar project, led by the Núcleo de Ecologia e Monitoramento Ambiental (NEMA, Centre for Ecology and Environmental Monitoring) at Universidade Federal do Vale do São Francisco (UNIVASF, University of Vale do São Francisco), focuses on restoring the Caatinga habitat, which is crucial for the survival of the critically endangered Spix's macaw. The project addresses not only ecological restoration but also integrates the local community by considering traditional practices and the socioeconomic realities of the region, aiming to create a sustainable model that supports both biodiversity and the livelihoods of the Caatinga's inhabitants (Kupferschmidt 2022).

We present morphophysiological data on the dispersal units of 100 taxa from the Caatinga, including nine functional seed traits (Saatkamp *et al.* 2019), 1000 seed weight, number of seeds per kilogram, germination, seed water content, plant growth form, endemism, and the extinction risk for each georeferenced record. Over nine years of data collection (2014 to 2023), more than 60 tonnes of native Caatinga dispersal units were gathered, forming 1981 seed lots (Figure 1). In this study, a 'seed lot' refers to the collection of seeds from a species over a one-week period. Each lot underwent laboratory processing and analysis, with the remaining seed mass constituting the seed lot (Brasil 2013). For each seed lot, the germination rate, water content, and 1000 seed weight were evaluated. During this period, longevity was assessed in 763 seed lots. Longevity information assists in monitoring stored material and developing storage strategies, such as lot validity and discarding unviable material (Davies *et al.* 2020). This contributes not only to the expanding body of knowledge on various aspects of the Brazilian semiarid flora, such as spatial distribution, dispersal syndromes, morphology of dispersal units, and methods for overcoming seed dormancy, but also to research proposing sustainable management strategies aimed at preserving SDTFW. It involves examining the role of these dispersal units in forest dynamics and identifying factors that affect dispersal and germination.

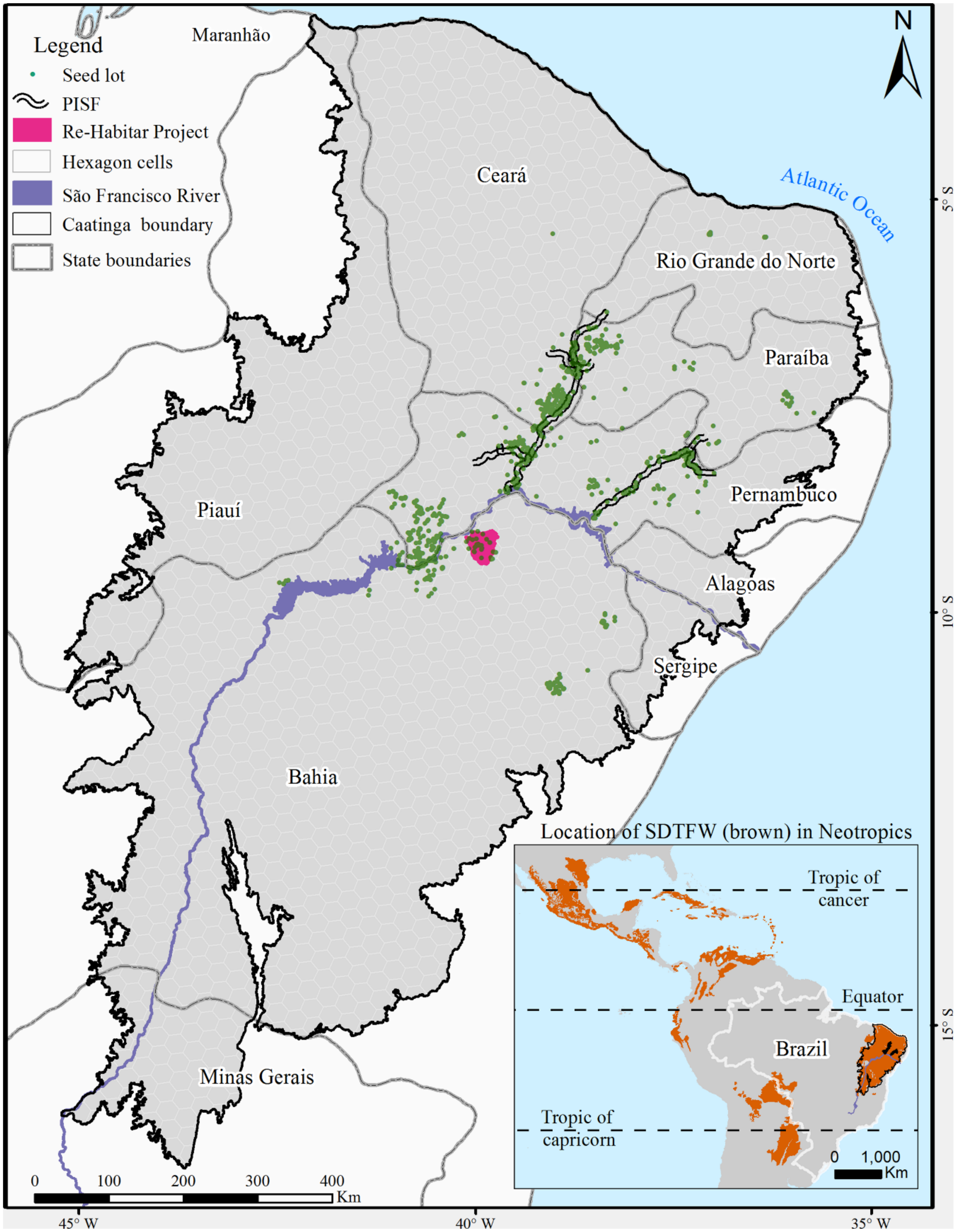

Figure 1. Distribution of seed lot records harvested by the Núcleo de Ecologia e Monitoramento Ambiental (NEMA) at the Universidade Federal do Vale do São Francisco (UNIVASF) in the seasonally dry tropical forest and woodland (SDTFW) in the Neotropics, covering the period from

2014 to 2023. The figure shows the geographic distribution of harvested seed lots across different regions within the SDTFW (Olson *et al.* 2001; DRYFLOR 2016; Queiroz *et al.* 2017). The collection of diaspores from native species of the Caatinga was conducted as part of the Projeto de Integração do Rio São Francisco (PISF, São Francisco River Integration Project), within the operational area of the RE-Habitar project, and in other strategically mapped areas to meet the demands of the PISF's degraded area recovery PBA.

Metadata S1

Class I. Data Set Descriptors

A. Data set identity:

Caatinga diaspores: A descriptive overview of dispersal units of seasonally dry tropical forests and woodlands

B. Data set identification code:

references.csv and traits_metadata.csv

C. Data set description

1. Originators:

Fabrício Francisco Santos da Silva, Núcleo de Ecologia e Monitoramento Ambiental, Campus Ciências Agrárias, Universidade Federal do Vale do São Francisco, Petrolina, Pernambuco, Brazil; fabriciofrancisco2006@gmail.com; ORCID: https://orcid.org/0000-0001-7938-8308

Edjane Silva Damasceno, Núcleo de Ecologia e Monitoramento Ambiental, Campus Ciências Agrárias, Universidade Federal do Vale do São Francisco, Petrolina, Pernambuco, Brazil; damasceno.des@gmail.com; ORCID: https://orcid.org/0000-0002-6325-8004

Ramon Athayde de Souza Cavalcante, Núcleo de Ecologia e Monitoramento Ambiental, Campus Ciências Agrárias, Universidade Federal do Vale do São Francisco, Petrolina, Pernambuco, Brazil; ramon-athayde@outlook.com; ORCID: https://orcid.org/0009-0007-8010-4967

Francinete Alves do Nascimento, Núcleo de Ecologia e Monitoramento Ambiental, Campus Ciências Agrárias, Universidade Federal do Vale do São Francisco, Petrolina, Pernambuco, Brazil; francinete.07alves@gmail.com; ORCID: https://orcid.org/0000-0002-3707-4156

Mateus Brandão Prates, Núcleo de Ecologia e Monitoramento Ambiental, Campus Ciências Agrárias, Universidade Federal do Vale do São Francisco, Petrolina, Pernambuco, Brazil; mbprates@gmail.com; ORCID: https://orcid.org/0009-0004-9044-7902

Luís Francisco Mello Coelho, Núcleo de Ecologia e Monitoramento Ambiental, Campus Ciências Agrárias, Universidade Federal do Vale do São Francisco,

Petrolina, Pernambuco, Brazil; coelholf@yahoo.com.br; ORCID: https://orcid.org/0000-0001-6901-1267

Daniel Salgado Pifano, Centro de Estudos em Biologia Vegetal, Campus Ciências Agrárias, Universidade Federal do Vale do São Francisco, Petrolina, Pernambuco, Brazil; daniel.pifano@univasf.edu.br; ORCID: https://orcid.org/0000-0001-8361-7337

Renato Garcia Rodrigues, Núcleo de Ecologia e Monitoramento Ambiental, Campus Ciências Agrárias, Universidade Federal do Vale do São Francisco, Petrolina, Pernambuco, Brazil; renato.garcia@univasf.edu.br; ORCID: https://orcid.org/0000-0001-5576-8621

2. Abstract: Dispersal unit characteristics provide crucial insights into species ecology and are essential for the conservation and restoration of ecosystems. The Caatinga, the largest ecosystem of Seasonally Dry Tropical Forests and Woodlands in South America, remains underrepresented in terms of dispersal unit data, which are often scattered across the scientific literature or remain unpublished. To address this gap, we compiled a dataset of morphophysiological data for 100 native taxa, including key information such as germination, seed water content, 1000 seed weight, fruit shape, and the geographic coordinates of 1981 seed lots. Over nine years, more than 60 tonnes of dispersal units were harvested, representing 91% of the most dominant woody species in this ecosystem. These records stem from environmental licensing actions associated with the São Francisco River Integration Project (PISF), the Re-Habitar Ararinha Azul Project, and verified literature. This dataset, the first of its kind for the Caatinga, offers valuable potential for research on forest dynamics, dispersal, germination, conservation, and ecological restoration in the Brazilian semiarid region. We hope this data paper provides reliable information on local flora distribution, dispersal syndromes, and morphological descriptions, while also suggesting methods for overcoming seed dormancy in the Caatinga. No copyright restrictions apply to this dataset, but please cite this data paper in publications. We also encourage researchers and educators to inform us of how they are using the data.

Resumo: As características das unidades de dispersão podem fornecer insights valiosos sobre a ecologia das espécies e suas implicações para a conservação e restauração de ecossistemas. A Caatinga é o maior ecossistema de Florestas e Bosques Tropicais Sazonalmente Secos da América do Sul. No entanto, as informações sobre as unidades de dispersão da região semiárida brasileira ainda são insuficientes e dispersas na literatura científica ou em dados não publicados. Para preencher essas lacunas e fornecer informações detalhadas sobre as unidades de dispersão da Caatinga, compilamos um conjunto de dados morfofisiológicos de 100 táxons nativos, incluindo informações como germinação, teor de água das sementes, peso de 1000 sementes, forma do fruto e coordenadas geográficas de 1981 lotes de sementes. No total, mais de 60 toneladas de unidades de dispersão foram coletadas, cobrindo 91% das espécies lenhosas mais dominantes neste ecossistema. Esses registros provêm de resultados parciais de ações de licenciamento ambiental do Projeto de Integração do Rio São Francisco com Bacias Hidrográficas do Nordeste Setentrional (PISF), do projeto RE-Habitar Ararinha Azul e de literatura confiável. Nosso conjunto de dados é o primeiro do tipo para a Caatinga e tem potencial para uso em estudos sobre dinâmica florestal, dispersão, germinação, conservação e restauração ecológica da região semiárida brasileira. Portanto, esperamos que este artigo de dados possa oferecer informações valiosas e confiáveis sobre a distribuição da flora local, síndromes de dispersão, descrições morfológicas e sugerir diferentes métodos para superação de dormência de sementes na Caatinga. Não há restrições de direitos autorais associadas ao uso deste conjunto de dados. Pedimos, por favor, que citem este artigo de dados ao utilizar as informações em publicações. Também solicitamos que pesquisadores e educadores nos informem sobre como estão utilizando os dados.

D. Key words/phrases: endemism; *ex situ*; SDTFW; seed collection; seed dormancy; seed longevity; seed mass; seed shape; seed storage; seed traits.

Palavras-chave: endemismo; endemismo; *ex situ*; SDTFW; coleta de sementes; dormência de sementes; longevidade de sementes; massa de sementes; forma de sementes; armazenamento de sementes; traços funcionais de sementes.

Class II. Research origin descriptors

A. Overall project description

1. Identity: Caatinga diaspores data set
2. Originators: The 'Caatinga diaspores data set' was coordinated by Fabrício Francisco Santos da Silva and Edjane Silva Damasceno, who were responsible for data standardisation and metadata writing. The review of taxonomic nomenclature according to 'Flora do Brasil' (BFG 2022) and additional metadata writing were collaboratively undertaken by Ramon Athayde de Souza Cavalcante, Francinete Alves do Nascimento, Mateus Brandão Prates, Luís Francisco Mello Coelho, Daniel Salgado Pifano and Renato Garcia Rodrigues.
3. Period of study: The data presented were harvested from 2014 to 2023. The process to organise and produce the current data set took place in 2023.
4. Objectives: Supply morphophysiological information on Caatinga diaspores.
5. Abstract: See section Class I. C. 2. Abstract.
6. Sources of funding: Fundings were provided by the Brazilian government from the Ministério da Integração e Desenvolvimento Regional (MIDR) as part of the Projeto São Francisco environmental licensing requirements. We also thank all team of the NEMA/UNIVASF and Centro de Estudos em Biologia Vegetal (CEBIVE) by support.

B. Specific subproject description

1. Site description: The Caatinga represents the largest and most continuous area of seasonally dry tropical forests and woodlands in South America, characterized by high levels of endemism (Queiroz *et al.* 2017; Fernandes *et al.* 2022). It harbours 3,347 species of flowering plants, of which 15% are endemics (Fernandes *et al.* 2020). The species exhibit typical adaptations to semiarid environments such as leaf drop during the dry period and water storage strategies in roots and stems

(Queiroz 2009; Moro *et al.* 2015). The vegetation composition differs mainly due to the aridity index (Silva and Souza 2018; Oliveira *et al.* 2022). The predominant climate is hot and dry (BSh), belonging to the semiarid morphoclimatic domain with low latitude and altitude, according to the Köppen climate classification. The mean annual temperatures vary between 23.4 and 24.8 ° C and the mean annual precipitation ranges from 541 to 627 mm. The rainy season occurs between the months of November and April (Alvares *et al.* 2013; INMET 2023).

In this data paper, we compile morphophysiological data from 1,981 seed lots of native species harvested across 83 municipalities in five states within the central region of the Caatinga. The total sampling area was calculated in two ways: first, by covering 116 hexagons where collections occurred, amounting to 6,789,290 hectares; second, by considering the municipalities themselves, with a total area of 11,432,716 hectares. The seed lots were harvested from sites with distances ranging from 0 km (indicating collections from the same site) to a maximum of 802 km between the furthest collection locations (Figure 1).

2. Research methods

To address the need for seed rescue and seedling production for the restoration of areas degraded by the project's installation, the NEMA/UNIVASF research center established the Seeds Network of the São Francisco River Integration Project in 2015. This network aims to support environmental efforts related to PISF licensing, strengthen the native seedling production chain in the project region, and assist Brazilian scientific research by donating seeds and providing technical support to partner nurseries (Damasceno *et al.* 2021; NEMA 2023; Padovezi *et al.* 2024).

Each seed lot harvested for the PISF Seeds Network represents seeds from a species gathered over a one-week period. After collection, the seeds undergo extraction, processing, and physical characterization in the laboratory. The remaining seed mass after processing forms the seed lot, which is assigned a unique identification code (Brasil 2013). All related data are recorded in a purpose-built database, ensuring full traceability and data summarization. This data paper compiles both published and unpublished information on dispersal units from the largest SDTFW

in the Neotropics—the Caatinga. The primary data come from nine years of seed rescue and research by NEMA/UNIVASF, conducted as part of the PISF Degraded Area Recovery Plans and Basic Environmental Plans (PBA) 23, both coordinated by UNIVASF (Rodrigues *et al.* 2019; Damasceno *et al.* 2021). Additionally, the RE-Habitar Ararinha Azul Project (Damasceno *et al.* 2022; Kupferschmidt 2022; Vercillo *et al.* 2023), undertaken by NEMA/UNIVASF in Juazeiro and Curaçá, Bahia, also contributes to the presented data (Figure 2).

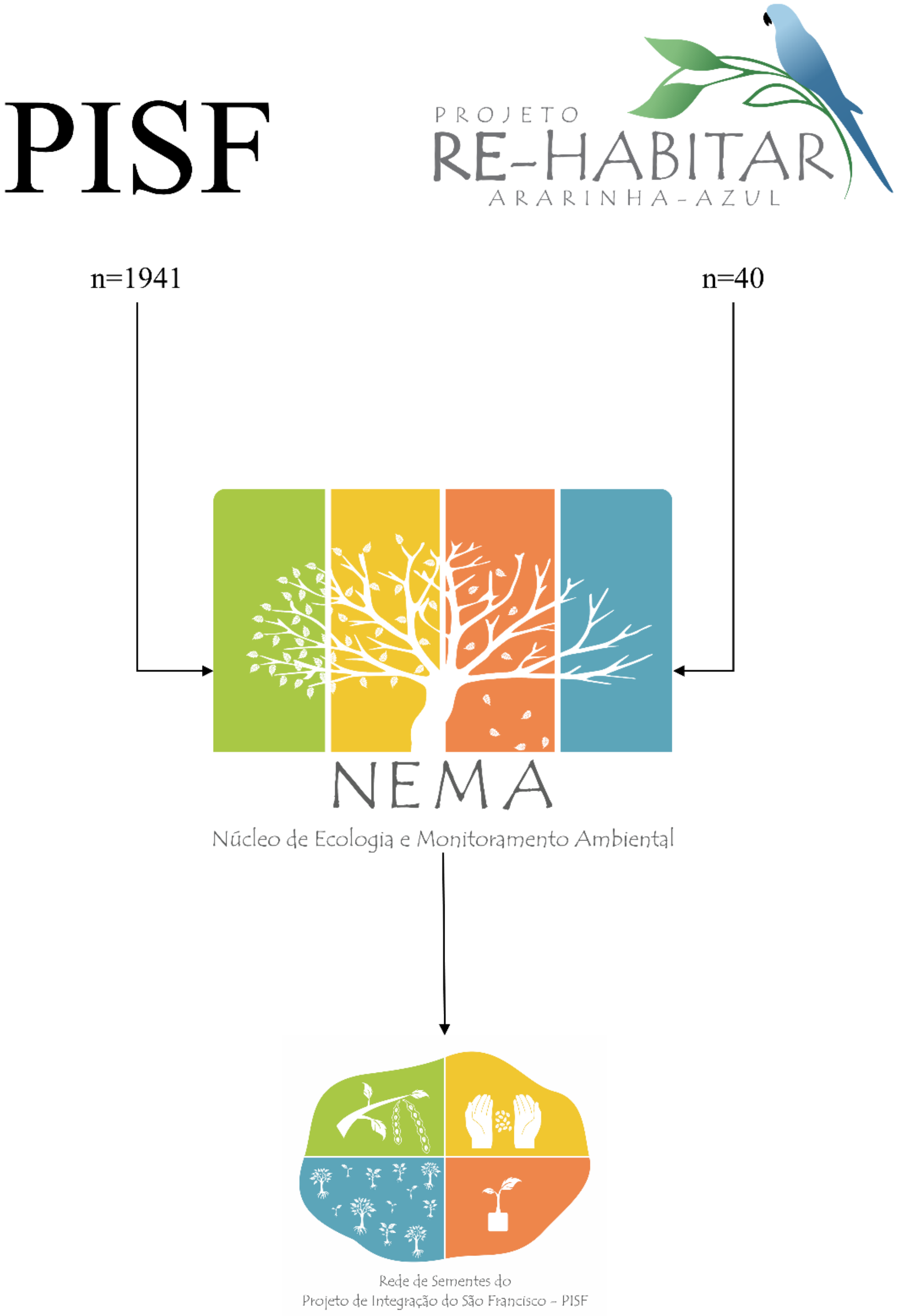

Figure 2. Over nine years of data collection (2014 to 2023), more than 60 tonnes of native Caatinga dispersal units were harvested, resulting in 1981 seed lots. The seeds were obtained through seed collection and germplasm rescue activities

carried out under the Projeto de Integração do Rio São Francisco (PISF, São Francisco River Integration Project) and the Projeto Re-Habitar Ararinha Azul (Re-Habitar Ararinha Azul Project), both executed by the Núcleo de Ecologia e Monitoramento Ambiental (NEMA) at the Universidade Federal do Vale do São Francisco (UNIVASF). The PISF is one of the largest water infrastructure projects globally, funded by the Brazilian government's Ministério da Integração e Desenvolvimento Regional (MIDR, Ministry of Integration and Regional Development). The Re-Habitar Ararinha Azul Project is part of the Global Environmental Facility (GEF) – Terrestrial portfolio, financially supported by the Inter-American Development Bank (IDB) and coordinated by the Ministério do Meio Ambiente (MMA, Ministry of the Environment) through the Fundo Brasileiro para Biodiversidade (FUNBIO, Brazilian Biodiversity Fund). Seed processing and analysis are conducted at NEMA. After these procedures, the seed lots are transferred to the Rede de Sementes do Projeto de Integração do São Francisco (PISF Seed Network), which aims to strengthen the native seedling production chain in the project region, while also assisting Brazilian scientific research by donating seeds and providing technical support to partner nurseries. Logotypes of the Re-Habitar Ararinha Azul Project, NEMA, and the PISF Seed Network, owned by Renato Garcia Rodrigues©.

Over 80 staff members, from both scientific and administrative sectors, have been directly involved in these projects. The infrastructure includes key facilities like the Ecology Laboratory, Multi-purpose Laboratory, Geoprocessing Laboratory, Seed Processing Laboratory, two greenhouses, and a well-organized administrative sector that coordinates logistics and research activities. This collective effort has enabled the efficient execution of large-scale seed collection, germplasm rescue, and restoration projects, resulting in a significant environmental impact.
The collection of diaspores from native Caatinga species occurred in the direct influence area of the PISF, the operational area of the RE-Habitar project, and other strategically mapped regions to meet the recovery demands of the PISF's degraded

areas as outlined in the PBA. Taxonomic identification of the harvested material was supported by the Herbário de Referência do Sertão Nordestino (HRSN).

Seed collection efforts were adjusted based on the accessibility and ecological characteristics of each area. Field campaigns typically lasted between 7 to 15 days, depending on logistical challenges and the size of the region being sampled. Collections were often conducted along pre-established trails of 1 to 3 km in length, with teams of 6 to 10 collectors per campaign working collaboratively to ensure thorough coverage and efficiency. The frequency of collections varied by location, with some sites being sampled annually to monitor fluctuations in seed production over time.

After collection, diaspores were placed in nylon bags and transported to NEMA/UNIVASF for seed extraction and processing. Extraction was performed manually or mechanically depending on the fruit characteristics (Figure 3). During processing, immature, deteriorated, or damaged seeds were removed to ensure the quality of the seed lot.

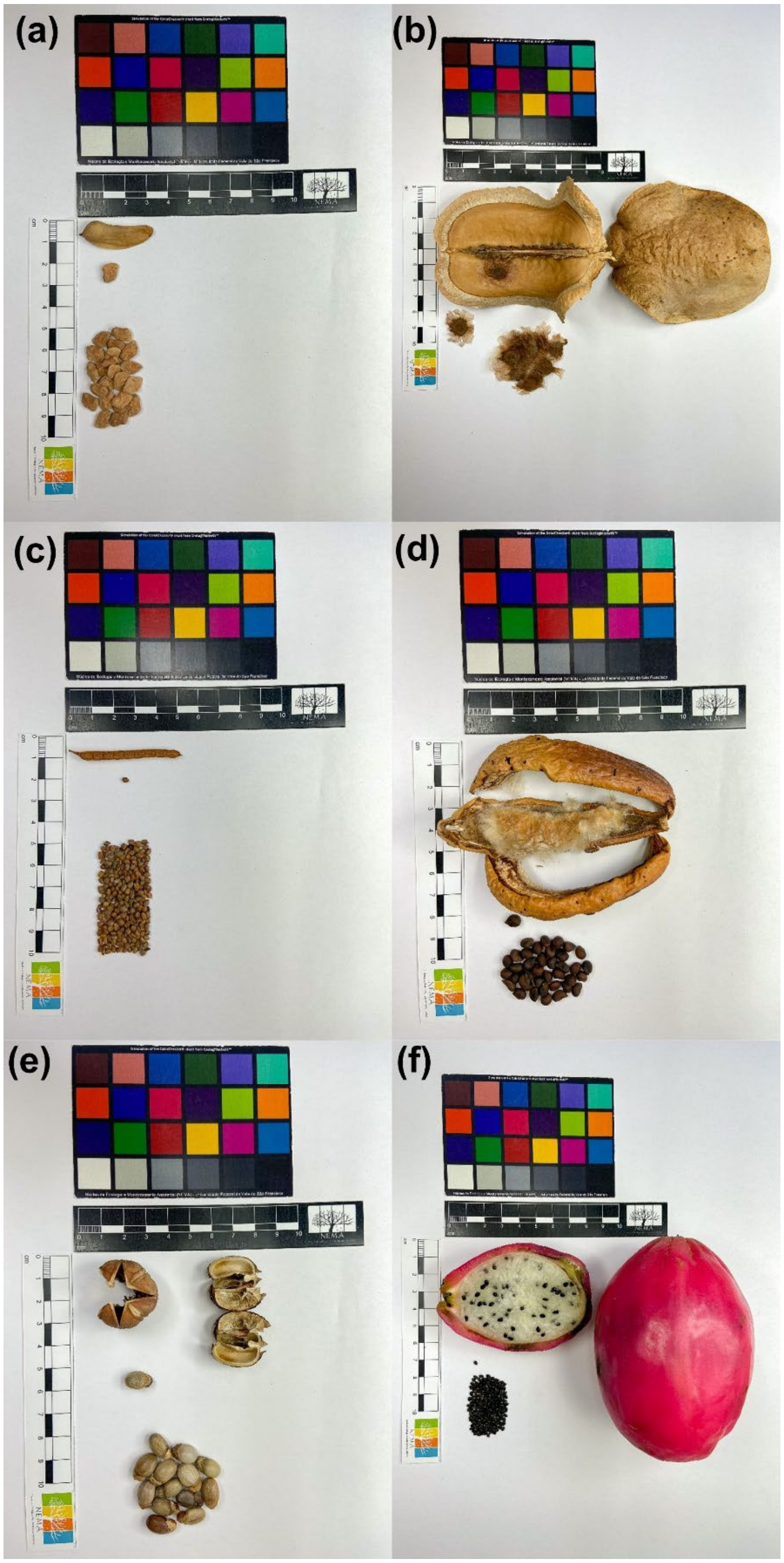

Figure 3. Fruit and seed types harvested: dry, indehiscent, winged fruits of *Schinopsis brasiliensis* Engl. – Anacardiaceae (a); dry, dehiscent fruit and winged seeds of *Jacaranda brasiliana* (Lam.) Pers. – Bignoniaceae (b); dry, dehiscent

fruits and diaspores of *Senna uniflora* (Mill.) H.S.Irwin & Barneby – Fabaceae (c), *Ceiba glaziovii* (Kuntze) K.Schum. – Malvaceae (d) and *Jatropha mollissima* (Pohl) Baill. – Euphorbiaceae (e); fleshy fruit and seeds of *Cereus jamacaru* DC. – Cactaceae (f). Images (a–f) by Renato Garcia Rodrigues© 2023. NEMA logotype, owned by Renato Garcia Rodrigues©.

a. Morphological Analysis of Fruits and Seeds

The physiological characteristics of the seeds were assessed based on their germination percentage. In the physical characterization of seed lots, several parameters were measured, including seed water content (%), 1000 seed weight, and seed mass at the time of collection (g). Understanding the water content of dispersal units helps determine their level of maturation and can influence the preservation of their physiological and sanitary integrity during storage (Ellis 2019).

The 1000 seed weight provides an estimate of seed size, maturity, and health (Brasil 2009), This parameter is also used to calculate sowing density, the number of seeds per seed lot, and the working sample weight required for laboratory analysis. The seed mass remaining after processing and laboratory analysis constitutes the seed lots.

To determine seed water content, the oven-drying method was used at 105 ± 3 °C for 24 hours (Brasil 2009, 2013). The 1000 seed weight was calculated from eight samples, each containing 100 seeds (Brasil 2009).

b. Seed Physiology Analysis

To assess germination capacity, germination tests were conducted in a biochemical oxygen demand (BOD) germination chamber, with a 12-hour photoperiod. Seed sterilization was carried out using sodium hypochlorite (2.0 to 2.5% w/v of active chlorine) at a concentration of 0.5% v/v. For each seed lot, four replications of 25 seeds were used. The seeds were placed either in double-lined Petri dishes with filter paper or sown on two moistened paper towels (Germitest®). The substrates were moistened with distilled water at a proportion of 2.5 times the dry weight of

the paper, and the seeds were kept at a constant temperature of 25 °C in the BOD chamber. Germination was counted once stabilization was observed, and root protrusion was used as the criterion for determining germination (Brasil 2009, 2013). For species with delayed germination, germination tests were conducted in plastic tubes with a substrate in nurseries. Some seeds underwent pre-germination treatments, such as the application of gibberellic acid (GA_3), 20-day imbibition, heating, partial or total removal of the endocarp, and mechanical scarification.

After germination tests, seeds were weighed and stored in various types of packaging, depending on the species (e.g., nylon bags, paper bags, plastic bags, plastic drums), and placed in a cold chamber at 8 °C. Seed lots stored in permeable packaging were periodically assessed for longevity, focusing on germination rate and seed water content. Most seed lots were evaluated after one year, with exceptions for three herbaceous species — *Senna uniflora* (Mill.) H.S.Irwin & Barneby (Fabaceae) and *Mesosphaerum suaveolens* (L.) Kuntze (Lamiaceae), which were evaluated every 6 months, and *Rhaphiodon echinus* (Nees & Mart.) Schauer (Lamiaceae), which was evaluated every 3 months. For further details, please refer to the traits_metadata.csv file.

The results from these evaluations were critical in determining whether seed lots would be designated for long-term storage, research, or direct planting. This information, combined with data on collection sites and the phenological status of parent plants, directs future collection efforts and optimizes seed acquisition strategies.

3. Project personnel:

Renato Garcia Rodrigues: coordinates NEMA/UNIVASF;

Daniel Salgado Pifano: coordinates CEBIVE and is the curator of HRSN;

Edjane Silva Damasceno: PISF Seed Network Researcher at NEMA/UNIVASF;

Ramon Athayde de Souza Cavalcante: Planning Manager responsible for seed collection reception and processing at NEMA/UNIVASF;

Fabricio Francisco Santos da Silva: researcher in charge of the Ecology Laboratory at NEMA/UNIVASF.;

Francinete Alves do Nascimento: environmental analyst in charge of the morphophysiological characterization of seeds at the Ecology Laboratory of NEMA/UNIVASF.;

Luís Francisco Mello Coelho: director of research and planning responsible for the geoprocessing department at NEMA/UNIVASF.

Mateus Brandão Prates: planning manager responsible for information systems and database at NEMA/UNIVASF.

Class III. Data set status and accessibility

A. Status

1. Latest update: July 5th, 2023
2. Latest archive date: July 5th, 2023
3. Metadata status: Last update, December 18, 2023. Published version
4. Data verification: All localities were checked for accuracy and precision. The last nomenclature verification was carried out on July 5th, 2023, by searching species accepted name in Flora e Funga do Brasil (http://floradobrasil.jbrj.gov.br/, last access July 5th, 2023)

B. Accessibility

1. Storage location and medium: The complete data and metadata are available as Supporting Information in Data S1. Data are also available in Mendeley Data at (https://doi.org/10.17632/8t6yh9rm26.2)
2. Contact persons: Renato Garcia Rodrigues, Laboratório de Ecologia do Núcleo de Ecologia e Monitoramento Ambiental, Campus Ciências Agrárias, Universidade Federal do Vale do São Francisco, Petrolina, Pernambuco, 56300-000, Brazil. renato.garcia@univasf.edu.br
3. Copyright restrictions: None.

4. Proprietary restrictions: Please cite this data paper when the data are used in publications. We also request that researchers and teachers inform us of how they are using the data

Class IV. Data structural descriptors

A. Data set file

1. Identity and size: DataS1.zip (126 KB), traits_metadata.csv (584.23 KB) and the list of references cited in the original publication (D.O.I.): references.csv, 35.25 KB
2. Format and storage mode: all text files (.csv) are included in a compressed folder (.zip)
3. Header information: See Table 1 and 2 in section B for column descriptions

B. Variable information

Table 1. Descriptions for the references.csv file containing information from reliable articles for each species, and the D.O.I. is cited in the last column.

**Variables**	**Description**
order	Accepted taxonomic Order name according to 'APG IV' (APG IV 2016)
family	Accepted taxonomic Family name according to 'Flora do Brasil' (BFG 2022)
genus	Accepted taxonomic genus name according to 'Flora do Brasil' (BFG 2022)
epithet	Accepted taxonomic taxon name according to 'Flora do Brasil' (BFG 2022), reported by the collector
commonname	Vernacular names
life_forms	Plant growth-form. Woody components: PalmTree; Shrub; Shrub,Liana/Scandent/Vine; Shrub,subshrubs; Shrub,tree; Shrub,tree,Liana/Scandent/Vine; Shrub,tree,subshrubs; Tree. Non-woody components: Dracenoid,subshrubs; Herb; Herb,subshrubs; Liana/Scandent/Vine; Liana/Scandent/Vine,subshrubs; Shrub,herb,subshrubs; Subshrubs; Succulent (Fernandes *et al.* 2020; BFG 2022).
endemism	ED: species endemism and NE: no endemic, according to 'Flora do Brasil' (BFG 2022)

RED_List_2022	Plant species in each extinction risk category (IUCN 2022) out of 100 taxa assessed. DD: Data Deficient; EN: Endangered; LC: Least Concern; NE: Not evaluated; NT: Near Threatened
MMA_2022	Plant species in each extinction risk category (MMA 2022) out of 100 taxa assessed. EN: Endangered; VU: Vulnerable
extinction_risk_category_2013	Plant species in each extinction risk category (Martinelli and Moraes 2013) out of 100 taxa assessed. EN: Endangered; LC: Least Concern; NE: Not evaluated; NT: Near Threatened; VU: Vulnerable
fruit_consistency	Fruit fleshy or dry
fruit_dehisces	Fruit dehiscent or indehiscent
winged_nonwinged	Fruit winged or non-winged
fruit_form	Fruit form: compressed, compressed-flat, cylindrical, depressed-globose, ellipsoid, elliptic, elliptic to rounded (square to obovate), elongated shape with dorso-ventral flattening, falcate, fusiform, globose, laterally reticulate (muticous to prominently aristate, the spines often retrorsely barbed), linear, linear (spirally twisted,longitudinally striate-costate), loculicidal (margin undulating), moniliform, obconic, oblong, oblong-campanulate tubes, oblong-ellipsoid, oblong-elliptic, oblong-falcate, oblong-orbicular, oblong-ovoid, obovate, obovoid, orbicular, oval, ovate to obovate in outline (apex mostly umbilicate,base attenuate to truncate), ovoid, pyriform, pyriform (suborbicular), rounded, short (stout), sigmoidal (linear spiraled,short stipitate), spheroid, straight (slightly falcate), subglobose, triangular
fruit_type	Fruit type: amphisarca, baccoid, berry, camara, camarium, capsule, caryopsis, ceratium, coccarium, craspedium, cryptoloment, diclesium, drupe, follicle, legume, loculicidal capsule, nuculanium, nutlet, pepo, pseudosamara, samara, schizocarp, septicidal capsule, sorosus (Spjut 1994; BFG 2022)
dispersal_syndrome	Species seed dispersal syndrome: anemochory; autochory; zoochory
seed_form	Seed form: angular (embedded in kapok), cochleariform (flattened), compressed-globose (smooth and embedded in kapok), ellipsoid, elliptic, elliptical acuminate, flattened, flattened (winged wide-elliptical subtrapezoidal), flattened

	(winged), flattened-convex, fusiform, globose, hat-shaped (symmetric hilum-micropylar region expanded), lenticular, linear-oblong, oblanceolate, obliquely obovoid but laterally compressed and very slightly carinate, oblong, oblong (aril red), oblong (ellipsoid white arils that completely cover the seeds), obovate, obovate,obovate-oblong (winged), orbicular, orbicular (winged), oval, ovoid, ovoid (caruncule seed), ovoid-obliquely, pyramidal, reniform, reniform (with narrow sinus or ubcochleate and slightly twisted), rhombic, rounded, rounded (embedded in kapok), rounded to pyriform to reniform (embedded in kapok), rounded,oblong,reniform, sickle wings, subglobose, subglobose (carunculate seed), suborbicular (winged), subreniform, subsquares, testa with cuticular folds, triangular, triangular (ovoid)
storage_behaviour	ORT: Orthodox, REC: Recalcitrant, INT?: Intermediate? (SER *et al.* 2023) or when the species was found in previous published articles (in this case, we provided the reference for the article, D.O.I); ORT_NEMA: Orthodox, according to metadata Nema; REC_NEMA: Recalcitrant, according to metadata Nema
D.O.I.	Digital Object Identifier link related to each paper. When a D.O.I. was not available, a link or full reference was provided

Table 2. Descriptions for the traits_metadata.csv file containing information for each seed lot harvested during nine years of data collection by NEMA/UNIVASF, mainly in the areas of operation of the PISF.

**Variables**	**Description**
record_id	Identification code, unique for each seed lot. The seed lot is formed by collecting a species over the course of a week, during which the mass of seeds remaining after the laboratory processing and analysis will correspond to the seed lot (Brasil 2013)
order	Accepted taxonomic Order name according to ‘APG IV’ (APG IV 2016)

family	Accepted taxonomic Family name according to 'Flora do Brasil' (BFG 2022)
genus	Accepted taxonomic genus name according to 'Flora do Brasil' (BFG 2022)
epithet	Accepted taxonomic taxon name according to 'Flora do Brasil' (BFG 2022), reported by the collector
author	Author of the species name reported in the paper
commonname	Vernacular names
project	PISF Axes (Integration Project of the São Francisco River with Watersheds of the Northern Northeast). PRAD: Programa de Recuperação de Áreas Degradadas. RAG: Ramal do Agreste. RAP: Ramal do Apodi. Re-Habitar: Projeto Re-Habitar Ararinha-azul
matrix_kind	Matrix kind: matrix region; matrix; deforested area
localization	State and Municipality of the locality
latitude	Latitude of the study site (decimal degrees)
longitude	Longitude of the study site (decimal degrees)
elevation	Elevation above sea level (m)
collector	Fruit and seed collector
collect_date	Date when the diaspore was harvested (DD.MM.YYYY)
seasonality_of_seed_release	Period or time of the year seed realease takes place
fruit_mass	Fruit mass harvested (g)
initial_mass	Seed mass when the seed lot was harvested (g)
result_mass	Mass the seed lot in 31/01/2023 (g)
weight_in_1000	1000 seed weight (g)
seeds_per_kg	Number of seeds per kilogram
humidity	Mean seed water content (%)
germinability	Mean germination rate for newly harvested seed lot (%)
seed_handling	Seed dormancy class, PY: Physical dormancy – imbibition and mechanical scarification. PD: Physiological dormancy – heating and partial or total removal of the endocarp. PY+PD: Combinational dormancy – mechanically scarified + gibberellic acid (GA_3). ND: Nondormant (Rosbakh *et al.* 2020; *sensu* Baskin and Baskin 2021)
observations	Observations unique for each seed lot
storage	Packaging of seed: nylon bag; paper bag; plastic bag; plastic drum
germinability_history	Mean germination rate for each seed lot in storage times. Root protrusion was the criterion used to consider germination and

	germinability was calculated [G =(N/100)100, where N = number of seeds germinated at the end of test. Unit: % (Ranal and Santana 2006)].
humidity_history	Seed water content [Rules for Analysis of Seeds (RAS), Unit: % (BRASIL 2009)] for each seed lot in storage times
days_after_storage	The time (in days) between the storage date and the longevity tests mentioned in the 'inserted_in_system' and 'test_date_storage' columns.
test_date_storage	Date of germination and seed water content analysis (DD.MM.YYYY) for each seed lot in storage times
inserted_in_system	Date of data entry into the system

C. Data anomalies: Missing information was classified as 'NA'.

Supplemental descriptors

A. Data acquisition

1. Data forms or acquisition methods: the collected samples were entered into the project database, Flora (v. 9.0, PostgreSQL), created exclusively to store data from the actions of the Fauna and Flora Conservation PBA. Information on plant growth form, endemism, extinction risk category, diaspore structures, and dormancy overcoming methods were appropriately documented for each species (DOI [references.csv])
2. Location of completed data forms: the deployment forms were compiled into the spreadsheet 'traits_metadata.csv' and can be found in the data archive
3. To ensure that the digital dataset is free of errors, several verification procedures were implemented. Taxonomic family information, genus, scientific name, author of the species names, plant growth form, and endemism were cross-checked using the "Flora do Brasil" (BFG 2022). Outliers for thousand seed weight, seeds per kilogram, and water content were not considered (NA). An outlier detection method based on standard deviation was applied. The data were filtered by species, and the mean and standard deviation for each variable were calculated. Values exceeding

the threshold of [mean ± 3*(standard deviation)] were identified as outliers (Benhadi-Marín 2018). To enhance accuracy, sensitivity was fine-tuned to 5%, allowing values near the upper and lower limits to be visually reassessed. Outliers were evaluated only for species with more than five seed lots. In the case of *Rhaphiodon echinus*, seeds were stored in different structures and compared separately. Initially, seeds were extracted from globose fruits before storage. However, due to processing challenges, diaspore units containing multiple seeds were stored instead, leading to early lots with thousand seed weights below 5 grams. This method ensures consistency and accounts for the natural intraspecific variability.

B. Publications

Medeiros, A. S., Scaloppi, J. C., Damasceno, E. S., Goto, B. T., Vieira, D. C. M., Socolowski, F., Rodrigues, R. G., & Yano-Melo, A. M. (2023). Arbuscular mycorrhizal fungi communities shaped by host-plant affect the outcome of plant–soil feedback in dryland restoration. Journal of Applied Ecology, 60, 507–518. https://doi.org/10.1111/1365-2664.14330

Carvalho, J. N., Beckmann-Cavalcante, M. Z., Rodrigues, R. G., Fontana, A. P., & Pifano, D. S. (2022). Native Caatinga species for the recovery of degraded areas in the brazilian semiarid region. Revista Árvore, 46, 1-12. https://doi.org/10.1590/1806-908820220000010

Rodrigues, R. G., Socolowski, F., Vieira, D. C. M. (2022). Avanços na recuperação da Caatinga. Cadernos do Diálogo - Desafios para ganhar escala na restauração florestal e o papel da sociedade civil, v. 10, p. 42 – 45.

Lima, R. G. (2022). Determinação químico-bromatológica e de compostos fenólicos das sementes de *Senna uniflora* como potencial uso em sistema de endozoocoria por caprinos. Monografia (Bacharel em Ciências Biológicas) - Universidade Federal do Vale do São Francisco, Petrolina-PE, Brazil.

Damasceno, E. S., Lima, D. de J., & Rodrigues, R. G. (2021). Conhecimento ecológico da flora da Caatinga na região do Projeto de Integração do Rio São Francisco. Revista

Brasileira de Educação Ambiental (RevBEA), 16 (3), 237–255. https://doi.org/10.34024/revbea.2021.v16.10834

Socolowski, F., Vieira, D. C. M., Souza, B. R., Melo, F. P. L. & Rodrigues, R. G. (2021). Restauración de la Caatinga: métodos propuestos para recuperar el más exclusivo y menos conocido ecosistema de Brasil. Multequina, 30 (2), 247-263.

Medeiros, A. S. (2021). Produção de inoculante micorrízico e aplicação em plantas usadas na recuperação de áreas degradadas do Projeto de Integração do Rio São Francisco. Dissertação (Mestrado em Agronomia - Produção Vegetal) - Universidade Federal do Vale do São Francisco, Petrolina-PE, Brazil.

Carvalho, J. N., Cavalcante, M. Z. B., Carvalho, P. A., Pifano, D. S., & Rodrigues, R. G. (2020). Ecophysiology germination of *Senna uniflora* seeds: species for recovery degraded areas. Journal of Seed Science, 42, 1-11. https://doi.org/10.1590/2317-1545v42238498

Urquiza, N. G, Carvalho, J. N., Corrêa, C. E., Pimentel, L. B., Pifano, D. S., & Rodrigues, R. G. (2019). Guia de propágulos e plântulas da Caatinga. Petrolina: Cogito. 58 p.

Granja, G. P. (2018). Demanda energética de diferentes conjuntos mecanizados para implantação de modelos de recuperação de áreas degradadas. Dissertação (Mestrado em Engenharia Agrícola) - Universidade Federal do Vale do São Francisco, Juazeiro-BA, Brazil.

Vieira, D. C. M., Athayde, E. A., Socolowski, F., Sá, I. B., Kiill, L. H. P., Pereira, L. A., Kuhlmann, M., Drumond, M. A., Carvalhães, M. & Rodrigues, R. G. (2018). Época de coleta de frutos e sementes nativos para recomposição ambiental no bioma Caatinga. Brazilian Agricultural Research Corporation (EMBRAPA). https://doi.org/10.13140/RG.2.2.18331.41762

Jesus, A. R. S. (2017). Influência do estresse hídrico na germinação de sementes de cinco espécies nativas das Caatingas utilizadas para recuperação de áreas degradadas. Monografia (Bacharel em Engenharia Agronômica) - Universidade Federal do Vale do São Francisco, Petrolina-PE, Brazil.

Carvalho, J. N. (2016). Espécies nativas da Caatinga para recuperação de áreas degradadas: prospecção, ecofisiologia da germinação e crescimento de plantas. Dissertação (Mestrado em Agronomia - Produção Vegetal) - Universidade Federal do Vale do São Francisco, Petrolina-PE, Brazil.

Dantas, B. F., Passos, M. A. A. & Rodrigues, R. G. (2014). Rede de Sementes Florestais da Caatinga: histórico, ações e perspectivas. Informativo ABRATES, 24 (3), 80-83.
Socolowski, F., Vieira, D. C. M., Silva, F. F. S., & Rodrigues, R. G. (2014). Resgate de espécies endêmicas e ameaçadas de extinção da Caatinga. Informativo ABRATES, 24 (3), 68-72.

C. Results description:

The file references.csv provides the bibliographic references used to obtain information regarding each harvested species: life form; endemism; three categories of extinction risk classification (Martinelli and Moraes 2013; IUCN 2022; MMA 2022); dehiscence, fruit shape and type; dispersal syndrome; seed shape; dormancy and methods to overcome it; and seed storage behaviour. The file traits_metadata.csv contains information on all seed lots recorded in our database.

Between 31/01/2014 and 31/01/2023, 1981 seed lots from Caatinga species were harvested, representing 63.99 tonnes of harvested fruits, which yielded 23.58 tonnes of stored seeds or diaspores (Figure 4). Twenty-nine botanical families encompassing 100 species were identified, with the six families represented by the highest number of species being Fabaceae (40 species, 995 seed lots), Malvaceae (8 species, 49 seed lots), Anacardiaceae (5 species, 132 seed lots), Bignoniaceae (5 species, 73 seed lots), Euphorbiaceae (5 species, 69 seed lots), and Cactaceae (5 species, 27 seed lots).

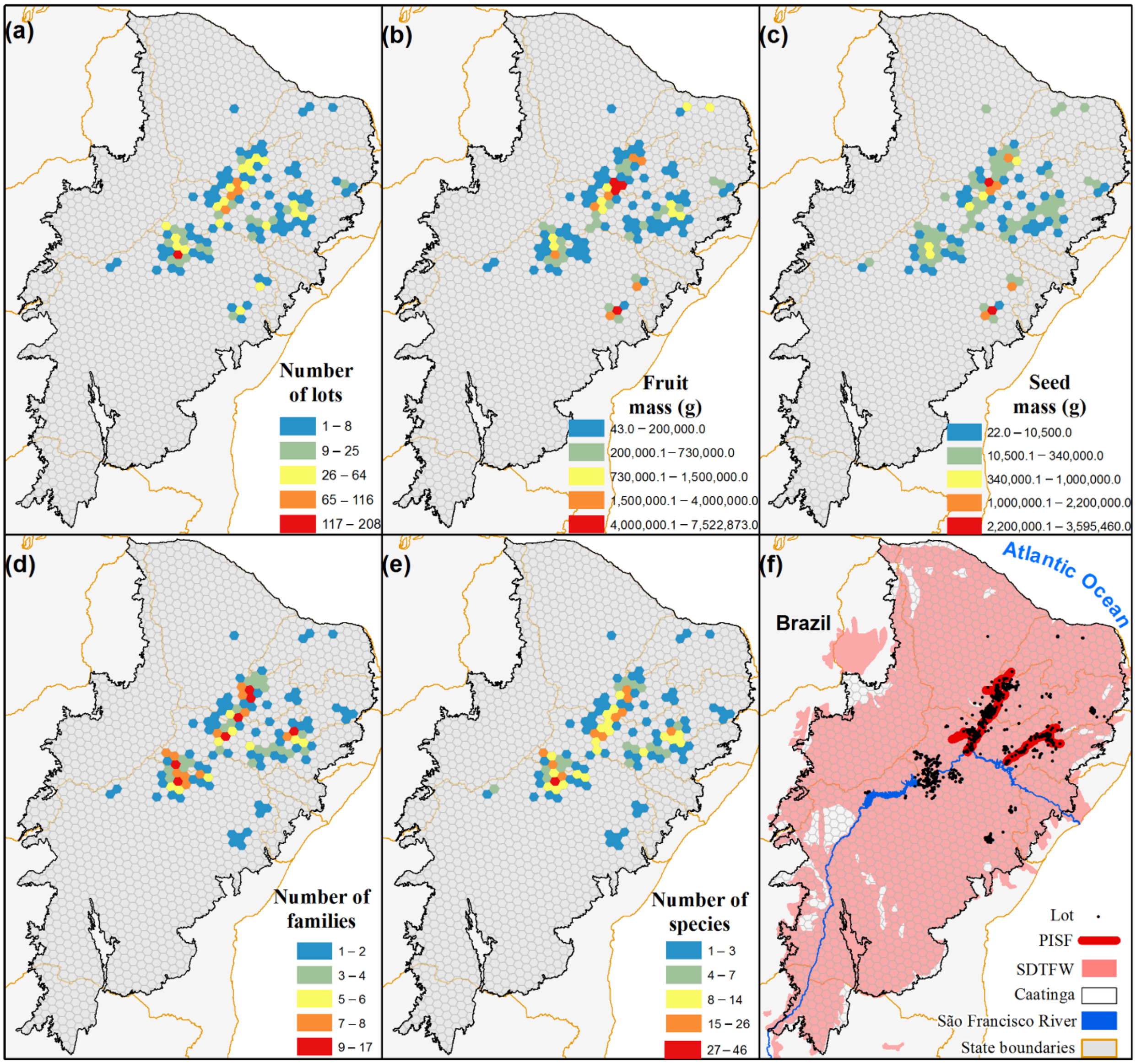

Figure 4. Spatial distribution and characteristics of 1981 seed lots harvested in the Caatinga, including areas influenced by the São Francisco River Integration Project (PISF), between 31/01/2014 and 31/01/2023: (a) Number of seed lots; (b) Fruit mass harvested (63,990,992.8 g); (c) Seed mass stored (23,582,563.51 g); (d) Number of botanical families (29); (e) Number of species (100); and (f) Collection points. This figure provides a detailed overview of the spatial distribution and key collection metrics, reflecting the diversity and scale of seed collection efforts in the Caatinga, a seasonally dry tropical forest and woodland (SDTFW).

Longevity was evaluated in 763 seed lots. This dataset allowed the determination of maximum storage times for species previously undocumented, such as *Bauhinia cheilantha* and

Copernicia prunifera. Information on seed lot longevity supports the development of storage strategies, including discarding non-viable material or monitoring stored material over time. Most stored seeds were classified as orthodox (74%). However, *Geoffroea spinosa*, *Inga vera*, and *Syagrus coronata* are recalcitrant species.

Most species (65%) exhibit some form of dormancy. Seed dormancy is a crucial evolutionary mechanism for seedling establishment and survival in seasonally dry forests such as the Caatinga (Souza *et al.* 2020). Given the significant diversity and complexity of primary dormancy mechanisms in seeds, classification through symbols/formulae helps to identify the cause of dormancy and, consequently, informs methods for breaking dormancy, such as treatment with gibberellic acid (GA_3) or warm and/or cold stratification (Baskin and Baskin 2021).

Information on fruit structure aids in understanding dispersal syndrome (Meiado *et al.* 2012). In this dataset, most species have legume-type fruits (20 species), followed by drupes (11 species) and loculicidal capsule (10 species). Of the 100 species analysed, 36 exhibit zoochoric dispersal syndromes (12 fruit types), 33 are autochoric (10 fruit types), and 31 are anemochoric (10 fruit types, with loculicidal capsule being the majority [8 species]). Most fruits are dry (72%), with all fruits exhibiting autochoric and anemochoric dispersal being dry, except for those of *Astronium* spp. The 26 species with fruits exhibiting zoochoric dispersal are divided into 16 species with fleshy fruits and 10 with dry fruits (Figure 5).

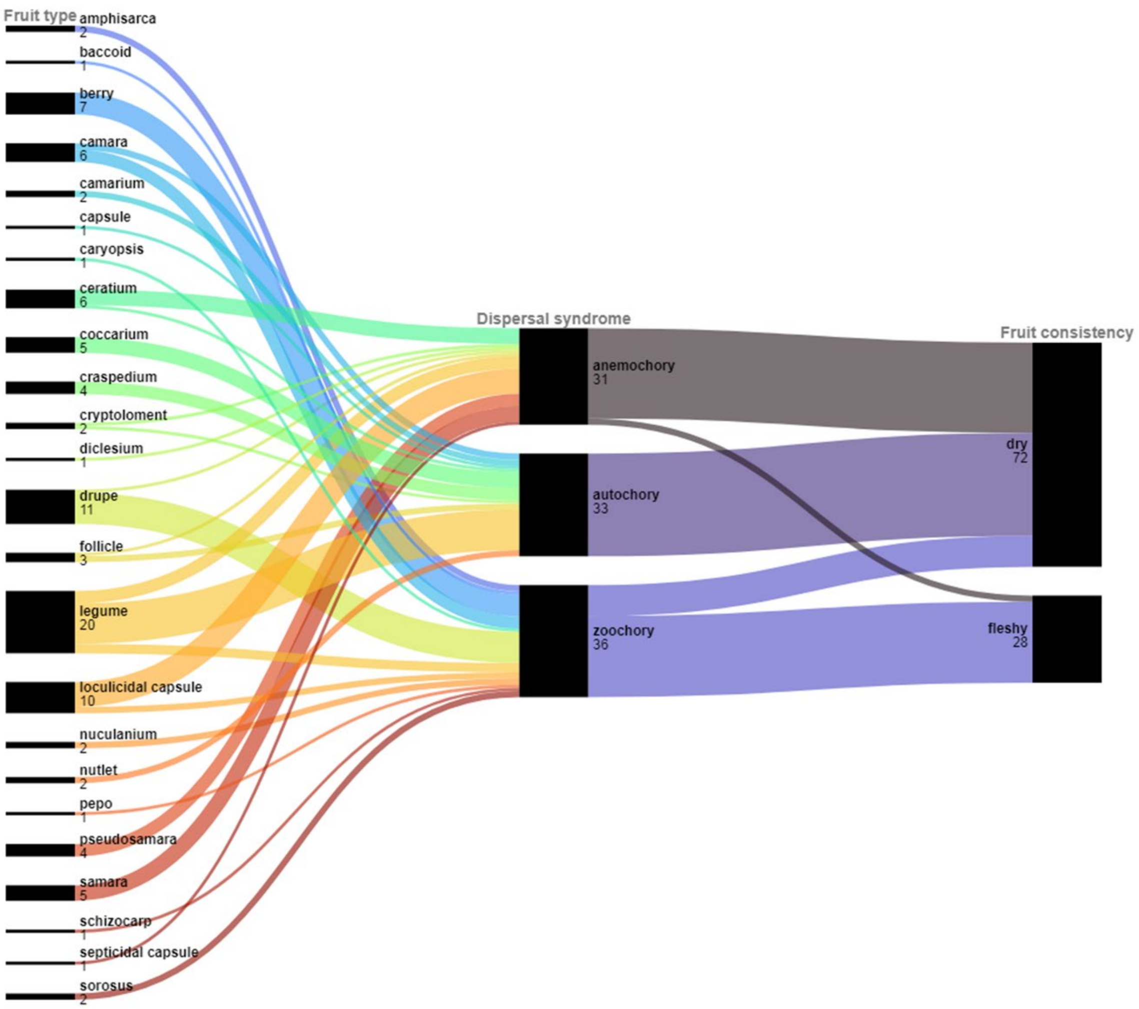

Figure 5. The Sankey chart categorically divides the number of species according to fruit type, dispersal syndrome, and fleshy or dry fruits, in that order. The chart visualises the relationships between fruit characteristics and dispersal mechanisms in the dataset, showing that most species have legume-type fruits (20 species), drupes (11 species) and loculicidal capsule (10 species). It highlights that most fruits are dry (72%) and that zoochoric dispersal is associated with both fleshy and dry fruits, whereas autochoric and anemochoric dispersal primarily involve dry fruits. The data was visualized using RAWGraphs (Mauri *et al.* 2017).

This dataset opens new possibilities for testing a wide range of hypotheses related to seed ecology, dispersal mechanisms, and restoration strategies in SDTFWs, such as the Caatinga. Hypotheses concerning seed dormancy, viability, and dispersal syndromes could be explored to deepen our understanding of how species adapt to varying climatic conditions and habitat

fragmentation. Furthermore, the data are crucial for guiding ecological restoration efforts by informing species selection based on viability, dormancy traits, and natural regeneration strategies.

Although comprehensive, the dataset reveals gaps in the representation of certain taxonomic and functional groups critical for ecosystem resilience. Future sampling efforts should aim to address these gaps, contributing to a more holistic understanding of the region's biodiversity and supporting long-term restoration success. In a context where seed databases are scarce (Liu *et al.* 2020; Rosbakh *et al.* 2020; Ordóñez-Parra *et al.* 2023), particularly those focused on the Caatinga (Fernández-Pascual *et al.* 2023), the relevance of this work becomes evident, highlighting its unique and significant contribution to understanding seed ecology in this ecosystem. Our database helps bridge the knowledge gap on seeds in the Caatinga, providing detailed information that can be utilised in studies on the ecology of woody species in seasonally dry environments.

D. History of data set usage

1. Data request history: N/A
2. Data set update history: Description of any updates performed on data set
3. Review history: N/A
4. Questions and comments from secondary users: N/A

Literature Citations

Alvares CA, Stape JL, Sentelhas PC, *et al.* 2013. Köppen's climate classification map for Brazil. *Meteorol Zeitschrift* **22**: 711–28.

Antongiovanni M, Venticinque EM, and Fonseca CR. 2018. Fragmentation patterns of the Caatinga drylands. *Landsc Ecol* **33**: 1353–67.

Antongiovanni M, Venticinque EM, Matsumoto M, and Fonseca CR. 2020. Chronic anthropogenic disturbance on Caatinga dry forest fragments. *J Appl Ecol* **57**: 2064–74.

APG IV. 2016. An update of the Angiosperm Phylogeny Group classification for the orders and families of flowering plants: APG IV. *Bot J Linn Soc* **181**: 1–20.

Baskin JM and Baskin CC. 2021. The great diversity in kinds of seed dormancy: A revision of the Nikolaeva-Baskin classification system for primary seed dormancy. *Seed Sci Res* **31**: 249–77.

Benhadi-Marín J. 2018. A conceptual framework to deal with outliers in ecology. *Biodivers Conserv* **27**: 3295–300.

BFG. 2022. Brazilian Flora 2020: Leveraging the power of a collaborative scientific network. *Taxon* **71**: 178–98.

Brasil. 2009. Regras para Análise de Sementes (RAS). Brasília: Mapa/ACS.

Brasil. 2013. Instruções para análise de sementes de espécies florestais. Brasília: Mapa/ACS.

BRASIL. 2009. Regras para análise de sementes. (Ministério da Agricultura Pecuária e Abastecimento. Secretária de Defesa Agropecuária, Ed). Brasília: Mapa/ACS.

Carvalho JN, Beckmann-Cavalcante MZ, Rodrigues RG, *et al.* 2022. Native Caatinga Species for the Recovery of Degraded Areas in the Brazilian Semiarid Region. *Rev Arvore* **46**: 1–12.

Cooper DLM, Lewis SL, Sullivan MJP, *et al.* 2024. Consistent patterns of common species across tropical tree communities. *Nature* **625**: 20–4.

Damasceno ES, Lima DJ, and Rodrigues RG. 2021. Conhecimento ecológico da flora da caatinga na região do Projeto de Integração do Rio São Francisco. *Rev Bras Educ Ambient*

16: 237–55.

Damasceno ES, Medeiros AS de, Martins CSG, *et al.* 2022. Sementes nativas da Caatinga: coleta, beneficiamento e armazenamento. Petrolina.

Dantas BF, Moura MSB, Pelacani CR, *et al.* 2020. Rainfall, not soil temperature, will limit the seed germination of dry forest species with climate change. *Oecologia* **192**: 529–41.

Davies RM, Hudson AR, Dickie JB, *et al.* 2020. Exploring seed longevity of UK native trees: Implications for ex situ conservation. *Seed Sci Res* **30**: 101–11.

Domingos-Melo A, Albuquerque-Lima S, Diniz UM, *et al.* 2023. Bat pollination in the Caatinga : A review of studies and peculiarities of the system in the new world ' s largest and most diverse seasonally dry tropical forest ☆. *Flora* **305**.

DRYFLOR. 2016. Plant diversity patterns in neotropical dry forests and their conservation implications. *Science (80-)* **353**: 1383–7.

Ellis RH. 2019. Temporal patterns of seed quality development, decline, and timing of maximum quality during seed development and maturation. *Seed Sci Res* **29**: 135–42.

Fernandes MF, Cardoso D, Pennington RT, and Queiroz LP. 2022. The Origins and Historical Assembly of the Brazilian Caatinga Seasonally Dry Tropical Forests. *Front Ecol Evol* **10**: 1–13.

Fernandes MF, Cardoso D, and Queiroz LP de. 2020. An updated plant checklist of the Brazilian Caatinga seasonally dry forests and woodlands reveals high species richness and endemism. *J Arid Environ* **174**: 1–8.

Fernandes MF and Queiroz LP. 2018. Vegetação e flora da Caatinga. *Cienc Cult* **70**: 51–6.

Fernández-Pascual E, Carta A, Rosbakh S, *et al.* 2023. SeedArc, a global archive of primary seed germination data. *New Phytol* **240**: 466–70.

Fonseca CR, Antongiovanni M, Matsumoto M, *et al.* 2017. Conservation opportunities in the Caatinga. Caatinga: The Largest Tropical Dry Forest Region in South America.

INMET. 2023. Rainfall INMET 1991–2020 (only in Portuguese).

https://portal.inmet.gov.br/normais. Viewed 26 Jul 2023.

IUCN. 2022. The IUCN Red List of Threatened Species. Version 2022-2. https://www.iucnredlist.org. Viewed 23 Aug 2023.

Kupferschmidt K. 2022. A wild hope. *Science (80-)* **376**: 1148–53.

Laumann PD, Ferreira MC, Silva DA, and Vieira DLM. 2023. Germination traits explain the success of direct seeding restoration in the seasonal tropics of Brazil. *For Ecol Manage* **529**.

Liu U, Cossu TA, Davies RM, *et al.* 2020. Conserving orthodox seeds of globally threatened plants ex situ in the Millennium Seed Bank , Royal Botanic Gardens , Kew , UK : the status of seed collections.

Manhães AP, Mazzochini GG, Marinho F, *et al.* 2022. Loss of plant cover mediates the negative effect of anthropogenic disturbance on the multifunctionality of a dryland. *Appl Veg Sci* **25**: 1–12.

Martinelli G and Moraes MA. 2013. Livro vermelho da flora do Brasil. (Andrea Jakobsson, Ed). Rio de Janeiro: Instituto de Pesquisas Jardim Botânico do Rio de Janeiro.

Mauri M, Elli T, Caviglia G, *et al.* 2017. RAWGraphs: A visualisation platform to create open outputs. *ACM Int Conf Proceeding Ser* 18–20.

Meiado MV, Silva FFS, Barbosa DCA, and Siqueira-Filho JA. 2012. Diaspores of the Caatinga: a review. In: Siqueira Filho JA de (Ed). Flora of the Caatingas of the São Francisco River: Natural History and Conservation. Rio de Janeiro.

MIDR. 2023. Projeto de Integração do Rio São Francisco. https://www.gov.br/mdr/pt-br/assuntos/seguranca-hidrica/projeto-sao-francisco. Viewed 14 Aug 2023.

MMA. 2022. PORTARIA MMA Nº 148, DE 7 DE JUNHO DE 2022. https://www.in.gov.br/web/dou/-/portaria-mma-n-148-de-7-de-junho-de-2022-406272733. Viewed 23 Aug 2023.

Moro MF, Macedo MB, Moura-Fé MM, *et al.* 2015. Vegetation, phytoecological regions and landscape diversity in Ceará state, northeastern Brazil. *Rodriguesia* **66**: 717–43.

NEMA. 2023. Núcleo de Ecologia e Monitoramento Ambiental.

https://nema.univasf.edu.br/sementes/. Viewed 14 Aug 2023.

Oliveira ACP de, Nunes A, Oliveira MA, *et al.* 2022. How Do Taxonomic and Functional Diversity Metrics Change Along an Aridity Gradient in a Tropical Dry Forest ? *Front Plant Sci* **13**: 1–9.

Oliveira ACP de, Nunes A, Pinho P, *et al.* 2020. From species presences to abundances: Using unevenly collected plant species presences to disclose the structure and functioning of a dryland ecosystem. *Ecol Indic* **113**: 1–7.

Olson DM, Dinerstein E, Wikramanayake ED, *et al.* 2001. Terrestrial Ecoregions of the World: A New Map of Life on Earth. *Bioscience* **51**: 933–8.

Ordóñez-Parra CA, Dayrell RLC, Negreiros D, *et al.* 2023. Rock n ' Seeds : A database of seed functional traits and germination experiments from Brazilian rock outcrop vegetation. *Ecology* **104**: 1–5.

Padovezi A, Adams C, Chazdon RL, *et al.* 2024. Native seed collector networks in Brazil: Sowing social innovations for transformative change. *People Nat* 1–17.

Queiroz LP. 2009. Leguminosas da Caatinga. Feira de Santana.

Queiroz LP, Cardoso D, Fernandes MF, and Moro MF. 2017. Diversity and Evolution of Flowering Plants of the Caatinga Domain. In: J.M.C. S, I.R. L, M. T (Eds). Caatinga. Cham.

Ranal MA and Santana DG. 2006. How and why to measure the germination process? *Rev Bras Bot* **29**: 1–11.

Rodrigues RG, Pifano DS, Socolowski F, *et al.* 2019. Técnicas de Seleção de Áreas e Metodologias de Recuperação Nota Técnica Nº 01/2019. Petrolina.

Rosbakh S, Baskin CC, and Baskin JM. 2020. Nikolaeva et al.'s reference book on seed dormancy and germination. *Ecology* **101**: 3049.

Saatkamp A, Cochrane A, Commander L, *et al.* 2019. A research agenda for seed-trait functional ecology. *New Phytol* **221**: 1764–75.

SER, INSR, RBGK, and SID. 2023. Seed Information Database (SID). https://ser-sid.org/.

Viewed 8 Aug 2023.

Shackelford N, Paterno GB, Winkler DE, *et al.* 2021. Drivers of seedling establishment success in dryland restoration efforts. *Nat Ecol Evol* **5**: 1283–90.

Silva AC and Souza AF. 2018. Aridity drives plant biogeographical sub regions in the Caatinga, the largest tropical dry forest and woodland block in South America. *PLoS One* **13**: 1–22.

Souza JD, Souza Aguiar BA, Santos DM, *et al.* 2020. Dynamics in the emergence of dormant and non-dormant herbaceous species from the soil seed bank from a Brazilian dry forest. *J Plant Ecol* **13**: 256–65.

Spjut RW. 1994. A systematic treatment of fruit types. New York.

Vercillo U, Oliveira-Santos LG, Novaes M, *et al.* 2023. Spix's Macaw Cyanopsitta spixii (Wagler, 1832) population viability analysis. *Bird Conserv Int* **33**: 1–12.
